# Comparative pathology of the nasal epithelium in K18-hACE2 Tg mice, hACE2 Tg mice, and hamsters infected with SARS-CoV-2

**DOI:** 10.1177/03009858211071016

**Published:** 2022-01-29

**Authors:** Pin Yu, Wei Deng, Linlin Bao, Yajin Qu, Yanfeng Xu, Wenjie Zhao, Yunlin Han, Chuan Qin

**Affiliations:** 1 Institute of Laboratory Animal Sciences, Chinese Academy of Medical Sciences (CAMS) & Comparative Medicine Center, Peking Union Medical College (PUMC); Beijing Key Laboratory for Animal Models of Emerging and Reemerging Infectious Diseases, Beijing, China

**Keywords:** SARS-CoV-2, pathology, olfactory epithelium, animal model, K8-hACE2 Tg mouse, hamster

## Abstract

Severe acute respiratory syndrome coronavirus-2 (SARS-CoV-2) causes severe viral pneumonia and is associated with a high fatality rate. A substantial proportion of patients infected by SARS-CoV-2 suffer from mild hyposmia to complete loss of olfactory function, resulting in anosmia. However, the pathogenesis of the olfactory dysfunction and comparative pathology of upper respiratory infections with SARS-CoV-2 are unknown. We describe the histopathological, immunohistochemical, and in situ hybridization findings from rodent models of SARS-CoV-2 infection. The main histopathological findings in the olfactory epithelia of K8-hACE2 Tg mice, hACE2 Tg mice, and hamsters were varying degrees of inflammatory lesions, including disordered arrangement, necrosis, exfoliation, and macrophage infiltration of the olfactory epithelia, and inflammatory exudation. On the basis of these observations, the nasal epithelia of these rodent models appeared to develop moderate, mild, and severe rhinitis, respectively. Correspondingly, SARS-CoV-2 viral RNA and antigen were mainly identified in the olfactory epithelia and lamina propria. Moreover, viral RNA was abundant in the cerebrum of K18-hACE2 Tg mice, including the olfactory bulb. The K8-hACE2 Tg mouse, hACE2 Tg mouse, and hamster models could be used to investigate the pathology of SARS-CoV-2 infection in the upper respiratory tract and central nervous system. These models could help to provide a better understanding of the pathogenic process of this virus and to develop effective medications and prophylactic treatments.

Coronavirus disease 2019 (COVID-19), caused by severe acute respiratory syndrome coronavirus-2 (SARS-CoV-2), has spread across the world and severely affected global health care systems.^32,40,41^ It commonly causes viral pneumonia, whereas multisystemic symptoms include headache, gastrointestinal symptoms, liver injury, and even olfactory and gustatory dysfunction.^7,21,22,38^ COVID-19 manifests as a mild respiratory syndrome in most individuals; however, severe cases are associated with a high fatality rate.^3,13,33^

The development of suitable animal models for COVID-19 would greatly assist in uncovering aspects of the pathogenesis and in evaluating the safety and efficacy of vaccine candidates, antibody candidates, or antiviral compounds. Previous studies have reported the features of a variety of animal models, including hamsters, nonhuman primates (macaques), mice, and ferrets,^1,4,16,29,36^ which supported the observations of viral replication in the lungs with mild to severe pathological changes. K18 human angiotensin-converting enzyme 2 (hACE2) transgenic (Tg) mice developed many features of severe COVID-19, including rhinitis and minimal mononuclear and neutrophilic interstitial pneumonia.^25^ Hamsters experimentally infected with SARS-CoV-2 exhibited diffuse alveolar damage and damage to multiple organs including the spleen, lymph nodes, different segments of the alimentary tract, kidneys, adrenal glands, ovaries, vesicular glands, and prostate.^28^

SARS-CoV-2 can attack the olfactory epithelium and olfactory nerves, resulting in olfactory dysfunction.^21,39^ Using the nasal epithelium as a portal, human coronavirus SARS-CoV-2 can travel along axons of the olfactory nerves from the nose to reach the brain.^5,6,24,31^ A better understanding of animal models is important for studying the pathogenesis of COVID-19 and for the preclinical evaluation of vaccines and antivirals against SARS-CoV-2 infection. Here, we describe the histopathological, immunohistochemical, and in situ hybridization (ISH) findings in K18-hACE2 Tg mice, hACE2 Tg mice, and hamsters infected with SARS-CoV-2. Although there have been animal model studies on the pathogenic mechanisms of SARS-CoV-2 infection in the lower respiratory system, little is known about the comparative pathology of the upper respiratory system. Therefore, it is vital to study the pathogenic mechanisms in the nasal passages in the commonly used animal models of SARS-CoV-2.

## Materials and Methods

### Ethics Statement

The safety and ethical aspects of this research on SARS-CoV-2 were discussed among the staff members of the Department of Pathogen Biology at the Institute of Laboratory Animal Science (ILAS) of the Chinese Academy of Medical Sciences and Peking Union Medical College (PUMC). The experiments and protocols for K18-hACE2 Tg mouse, hACE2 Tg mouse, and hamster models of SARS-CoV-2 infection were discussed explicitly and extensively among the staff members of the Department of Pathogen Biology. These discussions were followed by consultations with biosafety officers and facility managers at the ILAS of PUMC, as well as with numerous specialists in the fields of SARS-CoV-2 and general infectious disease research groups throughout China. All research procedures were approved by the ILAS Institutional Animal Care and Use Committee and the Laboratory Safety Committee (LSC). The approved registration numbers for this study are QC20010, BLL20001, and LJN20004. All experiments were conducted within an animal biosafety level 3 (ABSL-3) facility, which was constructed and accredited based on National Standard GB19489 at the ILAS of PUMC, Beijing, China.

### Study Design for the K18-hACE2 Transgenic Mouse Model of SARS-CoV-2 Infection

Three 8- to 10-week-old K18-hACE2 Tg mice of different sexes were provided by Gem Pharma tech Co., Ltd. The line of K18-hACE2 Tg mice was B6/JGpt-*H11*^em1Cin(K18-hACE2)^/Gpt, Strain No. T037657. The mice were infected with SARS-CoV-2 (accession number is MT093631.2, SARS-CoV-2/WH-09/human/2020/CHN) at 1 × 10^2^ TCID50 (50 µl per animal). Three K18-hACE2 Tg mice inoculated intranasally with an equal volume of phosphate buffered saline (PBS) were used as a mock control group. The animals were observed daily. After euthanasia at 3 days post-infection (dpi), tissue specimens including samples from lung, brain, and nasal cavity were collected for pathological evaluation.

### Study Design for the hACE2 Transgenic Mouse Model of SARS-CoV-2 Infection

Three 6- to 11-month-old hACE2 Tg mice of different sexes were inoculated intranasally with SARS-CoV-2 (the same accession number as above) at a dosage of 10^5^ TCID50 (50 µl per animal). Three hACE2 mice intranasally inoculated with an equal volume of PBS were used as a mock control group. The animals were observed daily, and the mice were euthanized at 5 dpi to collect lung, brain, and nasal cavity samples for histopathological analysis.

### Study Design for the Syrian Hamster Model of SARS-CoV-2 Infection

Syrian hamsters (*Mesocricetus auratus*) aged 4 to 6 months of different sexes were provided by Charles River. Six Syrian hamsters were inoculated intranasally with SARS-CoV-2 (the same accession number as above) at a dosage of 10^6^ TCID50 (100 µl per animal) and were used as the SARS-CoV-2 challenge group. Three hamsters inoculated intranasally with an equal volume of PBS were used as a mock control group (*N* = 3). The hamsters were observed daily. The animals were anesthetized prior to the procedures and, while under deep anesthesia, the animals were euthanized by exsanguination at 7 and 14 dpi (*N* = 3/day). Tissue specimens, including samples from lung, brain, and nasal cavity, were collected for pathological analysis.

### Histopathological Examination

The nasal cavities were fixed in 10% formalin solution and decalcified with 5% ethylenediaminetetraacetic acid (EDTA). Four transverse tissue levels of the nasal cavities were trimmed for observation of nasal passage epithelia, as follows. Tissue section 1 (T1) was taken immediately posterior to the upper incisor teeth and contained the proximal aspects of the nasal septum, nasal and maxillary turbinates, and the intranasal vomeronasal organ as its prominent morphologic features. Tissue section 2 (T2) was taken at the level of the incisive papilla and the duct that connects the nasal and oral cavities. Tissue section 3 (T3) was taken at the level of the second palatal ridge and contained the elaborate ethmoid turbinates and an intramural maxillary sinus adjacent to each nasal passage. Tissue section 4 (T4) was taken at the level of the first molar teeth and contained the cross-sectional profiles of the distal aspects of the nasal septum and ethmoid turbinates and the proximal nasopharyngeal meatus.^9,17^ The samples were dehydrated and embedded in paraffin according to conventional procedures, and 4-µm sections were prepared with a microtome. Some sections were stained with hematoxylin and eosin (HE) and periodic acid-Schiff (PAS) using routine methods.

### Immunohistochemistry

Briefly, serial sections were dewaxed and rehydrated in graded ethanol, and a standard avidin-biotin immunoperoxidase technique was performed.^12^ Table 1 lists the primary antibodies used for immunohistochemistry (IHC). Optimal antibody dilutions were determined in experiments with positive control tissues. Negative control sections were prepared using the same steps as described above, but the primary antibodies were derived from nonimmune serum.

**Table 1.** Primary antibodies used for immunohistochemistry.

Antibody	Major cells expressing/description	Source	Product number
SARS-CoV-2	SARS nucleocapsid protein	Abcam	ab273434
SARS-CoV-2	SARS-CoV-2 nucleocapsid protein	Abcam	ab273167
F4/80	Macrophage	Cell signaling technology	No.70076
Olfactory marker protein	Olfactory epithelium, olfactory bulb	Abcam	ab183947

Abbreviation: SARS-CoV-2, severe acute respiratory syndrome coronavirus-2.

### Dual IHC

Dual IHC for SARS-CoV-2 nucleocapsid protein and olfactory marker protein (OMP) was performed on the nasal cavities and olfactory bulb. After deparaffinization, the sections were exposed to 3% H_2_O_2_ for 5 minutes to quench endogenous peroxidase activity. Heat-induced epitope retrieval was performed in 10 mM citrate buffer, pH 6.0. After incubation for 25 minutes with 5% bovine serum albumin (BSA) to block nonspecific binding, the sections were immunolabeled using an anti-SARS nucleocapsid protein mouse monoclonal antibody (ab273434, Abcam) diluted 1:1000 and with an anti-olfactory marker protein rabbit monoclonal antibody (ab183947, Abcam) diluted 1:1000 in PBS. Sections were incubated overnight at approximately 4°C to allow antibody binding. After rinsing 3 times in PBS, the sections were incubated with an antirabbit IgG conjugated to alkaline phosphatase (AP) and an anti-mouse IgG conjugated to horseradish peroxidase (HRP) (DS-0003; ZSGB-BIO, China) at 37°C for 30 minutes, following the manufacturer’s instructions, and rinsed 3 times in PBS. Samples were then incubated with AP-red substrate at room temperature for 10 minutes. After rinsing 3 times in PBS, the samples were incubated with DAB at room temperature for 5 minutes in the dark. Then, the sections were rinsed with PBS 3 times prior to staining with hematoxylin for 5 minutes. Finally, the sections were mounted for observation and analysis.

### In Situ Hybridization

To examine SARS-CoV-2 genomic RNA in the lungs, brains, and nasal cavities, tissue sections were dewaxed and rehydrated in a graded ethanol series. ISH was carried out using the RNAscope2.5 HD Reagent Kit-RED (Advanced Cell Diagnostic [ACD], Cat. No. 322310) following the manufacturer’s instructions.^11,23^ Briefly, ISH Probe-V-nCoV2019-S (ACD, Cat. No. 848561, positive-sense RNA probe) (Genomic RNA fragment 21631-23303, RefSeq #NC_045512.2) was prepared and synthesized by ACD. After peroxidase blocking with 0.5% hydrogen peroxide at room temperature for 10 minutes, the sections were heated in antigen retrieval buffer and digested in the proteinase provided in the kit. Sections were incubated in ISH target probe at 40°C in a hybridization oven for 2 hours. After rinsing, the ISH signal was amplified using the preamplifier provided in the kit, amplifier-conjugated to AP, and incubated in DAB for visualization at room temperature. Then, the sections were stained with hematoxylin and mounted for observation and analysis.

### TUNEL Assay

To examine apoptosis in the nasal cavities, sections were prepared for the Terminal deoxynucleotidyl transferase (TdT) dUTP Nick-End Labeling (TUNEL) assay by means of the HRP-DAB TUNEL labeling kit (ab206386, Abcam), and the slides were counterstained with methyl green. Then, the sections were mounted for observation and analysis.

### Evaluation of Outcomes and Data Analysis

Lesion severity was scored according to their distribution and extent as follows: 0, no visible changes; 1, mild focal or multifocal changes; 2, moderate multifocal changes; 3, moderate diffuse change; 4, severe diffuse change. The intensity of the positive IHC or ISH signals in the nasal cavities was scored as follows: −, no detectable signal; +/−, weak or faint signal; + moderate, readily detectable signal; ++, strong, intense signal; +++, widespread signal. All evaluations were performed in a double-blinded manner.

## Results

### Pathological Findings in the Nasal Cavities of K18-hACE2 Transgenic Mice

**Figure 1.**
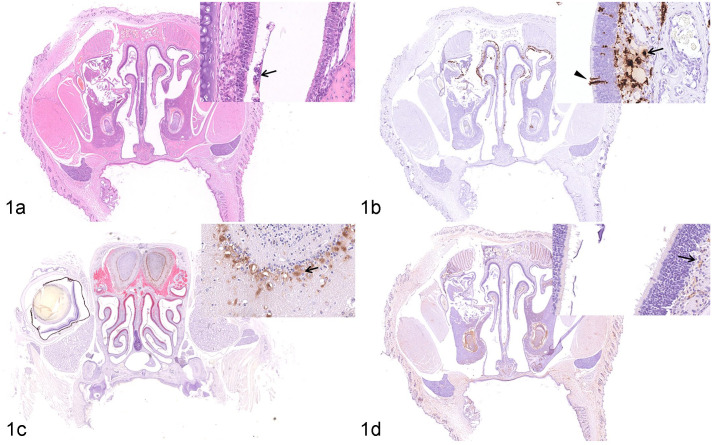
SARS-CoV-2 infection, nasal mucosa, K18-hACE2-transgenic (Tg) mouse, 3 days post-infection (dpi). (a) There is moderate rhinitis with focally disordered arrangement, necrosis, and sloughing (arrow) of the olfactory epithelium. Hematoxylin and eosin (HE). (b) Abundant viral RNA is detected in the olfactory epithelium (arrowhead) and in the lamina propria (arrow) underlying the olfactory epithelium. In situ hybridization (ISH) for SARS-CoV-2. (c) SARS-CoV-2 nucleocapsid protein is present in neurons of the olfactory bulb (arrow). Dual immunohistochemistry (IHC) for nucleocapsid protein (brown) and olfactory marker protein (red). (d) SARS-CoV-2 nucleocapsid protein is observed in the lamina propria (arrow) underlying the olfactory epithelium. IHC for nucleocapsid protein. Abbreviations: hACE2, human angiotensin-converting enzyme 2; SARS-CoV-2, severe acute respiratory syndrome coronavirus-2; IHC, immunohistochemistry.

The squamous, transitional, respiratory, and olfactory epithelia of the nasal passages of K18-hACE2 Tg mice were observed at 3 dpi (Table 2). The lesions were mainly found in the olfactory epithelia. Focal irregularity and necrosis of the olfactory epithelium were observed. A moderate amount of mucus was found primarily in the olfactory region of the nasal passage (Fig. 1a). In the respiratory epithelium, there was focal degeneration of respiratory epithelial cells and a small quantity of mucus on the epithelial surface. A large amount of viral RNA was detected by ISH in the olfactory epithelium and in the lamina propria underlying the olfactory epithelium (Fig. 1b). However, only moderate numbers of ISH-positive cells were detected in the respiratory epithelium. By IHC, SARS-CoV-2 nucleocapsid protein was principally present in the neurons of the olfactory bulb (Fig. 1c), in the olfactory epithelium, and in the lamina propria underlying the olfactory epithelium (Fig. 1d). No specific lesions related to experimental infection with SARS-CoV-2 were detected in the squamous epithelia and viral RNA was not detected.

**Table 2.** Lesions and viral RNA (in situ hybridization) and nucleocapsid immunolabeling in the nasal passages of K18-hACE2 Tg mice (*n* = 3), hACE2 Tg mice (*n* = 3), and hamsters (*n* = 3 per time point) infected with SARS-CoV-2.

		K18-hACE2 Tg mice	hACE2 Tg mice	Syrian hamsters	
		3 dpi	5 dpi	7 dpi	14 dpi
		Moderate	Mild	Severe	Mild
Epithelial lesion scores	Squamous	0	0	0	0
	Respiratory	1	0	3	0
	Olfactory	2	0	4	0
ISH (viral RNA)	Squamous	—	—	—	—
	Respiratory	+	—	+/−	—
	Olfactory	++	+/−	+	—
IHC (viral nucleocapsid)	Squamous	+	+/−	+	—
	Respiratory	++	+/−	++	—
	Olfactory	++	+/−	++	—

Lesion severity was scored by the distribution or extent of lesions as follows: 0, no visible changes; 1, mild focal or multifocal changes; 2, moderate multifocal changes; 3, moderate diffuse changes; 4, severe diffuse changes. The intensity of positive IHC or ISH signals in nasal cavities was scored as follows: −, no detectable signal; +/−, weak or faint signal; + moderate, readily detectable signal; ++, strong, intense signal.

Abbreviations: hACE2, human angiotensin-converting enzyme 2; Tg, transgenic; SARS-CoV-2, severe acute respiratory syndrome coronavirus-2; IHC, immunohistochemistry; ISH, in situ hybridization.

**Figures 2 and 3.**
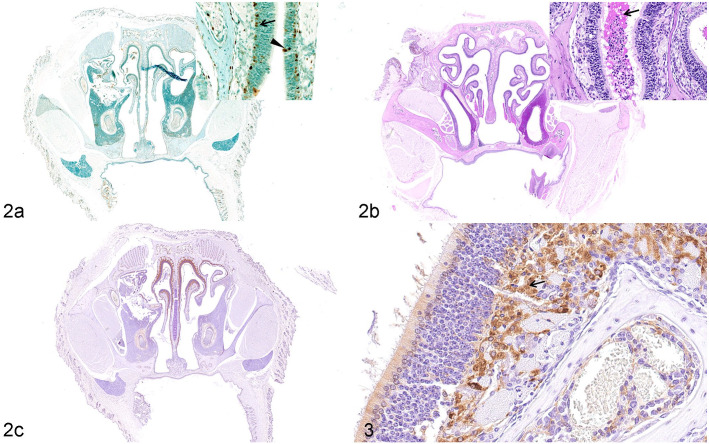
SARS-CoV-2 infection, nasal mucosa, K18-hACE2 Tg mouse, 3 days post-infection. (a) Apoptosis is detected in the basal cells (arrow) and nuclear layer of the olfactory sensory cells (arrowhead). TUNEL. (b) Mucous exudate (arrow) is observed in the nasal cavity. Periodic acid-Schiff. (c) and **Figure 3**. Many F4/80-positive macrophages (arrow, Fig. 3) infiltrate the lamina propria of the olfactory epithelium. Immunohistochemistry for F4/80. Abbreviations: SARS-CoV-2, severe acute respiratory syndrome coronavirus-2; hACE2, human angiotensin-converting enzyme 2; Tg, transgenic.

The pathogenesis of lesions in the olfactory epithelium was further investigated. Apoptosis of the basal cells and nuclear layer of the olfactory sensory cells was detected using TUNEL (Fig. 2a). Mucus exudation in the nasal cavity was confirmed by PAS reaction (Fig. 2b). A large number of F4/80-positive macrophages infiltrated the lamina propria of the olfactory mucosa (Figs. 2c, 3). Diffuse distribution of a small number of F4/80-positive macrophages was observed in lamina propria of the nasal passage of mock K18-hACE2 Tg mice (Fig. S3a). No significant pathological changes were observed in the counterpart tissues from the control K18-hACE2 Tg mice (data not shown; Supplemental Fig. S2–S3).

### Pathological Findings in the Nasal Cavities of hACE2 Transgenic Mice

**Figures 4 and 5.**
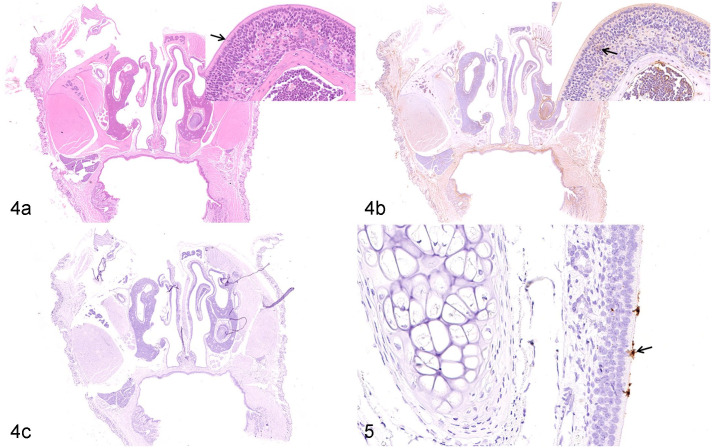
SARS-CoV-2 infection, nasal mucosa, hACE2 Tg mouse, 5 days post-infection. (a) The olfactory epithelium is intact (arrow, inset), and necrosis is not observed. Hematoxylin and eosin. (b) Faint immunolabeling for SARS-CoV-2 nucleocapsid protein is observed in the olfactory epithelium (arrow, inset). (c) and **Figure 5**. Viral RNA (arrow, Fig. 5) is scattered at the surface of the olfactory epithelium. In situ hybridization for SARS-CoV-2. Abbreviations: SARS-CoV-2, severe acute respiratory syndrome coronavirus-2; hACE2, human angiotensin-converting enzyme 2.

At 5 dpi, the squamous, transitional, respiratory, and olfactory epithelia of the nasal passages remained intact (Table 1). There were no significant necrotic lesions or inflammatory exudates in the olfactory epithelia (Fig. 4a). A small quantity of viral RNA was detected by ISH in the olfactory epithelia (Figs. 4c, 5), and faint IHC labeling for SARS-CoV-2 nucleocapsid protein was observed in the epithelia of the nasal passages (Fig. 4b). A few F4/80-positive macrophages were observed in the lamina propria of the olfactory epithelium. None of the significant pathological changes induced by SARS-CoV-2 infection were observed in the counterpart tissues from the control hACE2 Tg mice (data not shown; Supplemental Fig. S2–S3).

### Pathological Findings in the Nasal Cavities of Syrian Hamsters

**Figures 6 and 7.**
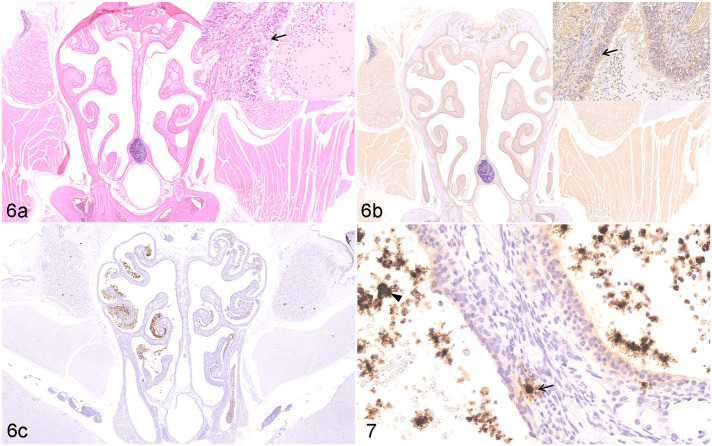
SARS-CoV-2 infection, nasal mucosa, Syrian hamster, 7 days post-infection. (a) There is disordered arrangement of olfactory epithelial cells (arrow, inset) and inflammatory exudation is observed. Hematoxylin and eosin (HE). (b) SARS-CoV-2 nucleocapsid protein (arrow, inset) is observed in the olfactory epithelium. IHC for nucleocapsid protein. (c) and **Figure 7**. Massive amount of viral RNA is detected in sloughed epithelial cells in the inflammatory exudate (arrowhead, Fig. 7) and a smaller quantity in the olfactory epithelium (arrow, Fig. 7). In situ hybridization (ISH). Abbreviations: SARS-CoV-2, severe acute respiratory syndrome coronavirus-2; IHC, immunohistochemistry.

At 7 dpi, the nasal cavity showed severe exudative rhinitis. Histologic lesions were extensive in the respiratory and olfactory epithelia. Degeneration of respiratory epithelium and lymphocyte infiltration of the respiratory mucosa was observed, along with a moderate number of inflammatory cells in the lamina propria. Mild exudate and inflammatory cells, primarily neutrophils, were focally distributed on the surface of the respiratory epithelium. The olfactory epithelial cells had a disordered arrangement, with necrosis and multifocal exfoliation of olfactory epithelial cells. Furthermore, a massive exudation of inflammatory cells, primarily neutrophils, and mucus filled the nasal cavity of the olfactory region. Numerous inflammatory cells infiltrated the lamina propria underlying the olfactory epithelium (Fig. 6a).

Viral RNA was diffusely detected in the olfactory epithelium and in the lamina propria underlying the respiratory epithelium. Furthermore, massive amounts of viral RNA were detected, using ISH, in the exfoliated cells within the inflammatory exudate, while a small quantity of viral RNA was multi-focally detected in the olfactory epithelium and the underlying lamina propria (Figs. 6c, 7). Diffuse SARS-CoV-2 nucleocapsid protein was diffusely detected using IHC in the epithelium of the nasal passage (Fig. 6b). No specific lesions related to experimental infection with SARS-CoV-2 or viral RNA distribution were detected on the squamous epithelia.

**Figures 8 and 9.**
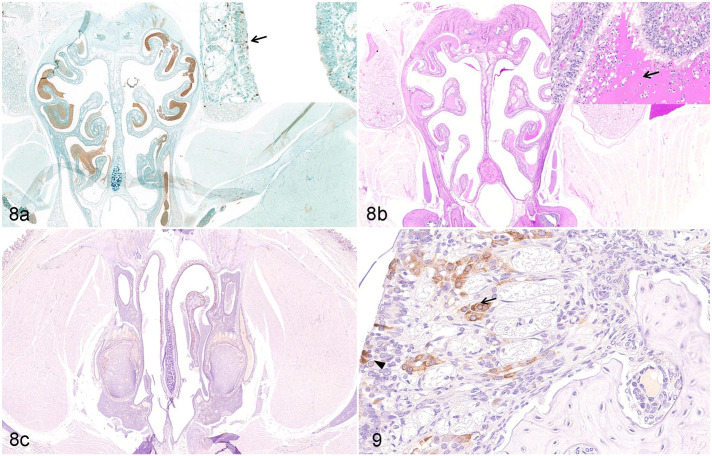
SARS-CoV-2 infection, nasal mucosa, Syrian hamster, 7 days post-infection. (a) Apoptosis is detected in the nuclear layer of sustentacular cells (arrow, inset) in the olfactory epithelium. TUNEL. (b) A large amount of mucus is accumulated on the surface of the olfactory epithelium in the nasal cavity (arrow, inset). Periodic acid-Schiff. (c) and **Figure 9**. F4/80-positive macrophages are detected in mucous layer (black arrowhead, Fig. 9) and submucosa (arrow, Fig. 9) of the necrotic olfactory epithelium. Immunohistochemistry (IHC) for F4/80. Abbreviations: SARS-CoV-2, severe acute respiratory syndrome coronavirus-2.

The pathogenesis of lesions in the olfactory epithelium was further investigated. Focal apoptosis was detected in olfactory epithelial cells using TUNEL (Fig. 8a). The nasal cavity had massive exudation of mucus that was PAS-positive (Fig. 8b). Numerous F4/80-positive macrophages infiltrated the lamina propria beneath the olfactory epithelium (Figs. 8c, 9). Diffuse distribution of a small number of F4/80-positive macrophages was observed in lamina propria of the nasal passage of mock-infected Syrian hamsters (Supplemental Fig. S3b). There were no significant pathological changes in the counterpart tissues from the control hamsters (data not shown).

The lesions in the nasal passage of the hamster model had mostly recovered at 14 dpi, and no obvious pathological changes were identified in the squamous, transitional, respiratory, or olfactory epithelia (Table 1). Although lesions in the olfactory epithelium returned to normal microscopically, mild disordered arrangement of the nuclear layer of the olfactory sensory cells was focally observed, and a minute quantity of filamentous exudation was observed on the surface of the olfactory epithelium (Supplemental Fig. S1a). No SARS-CoV-2 viral RNA (Supplemental Figs. S1c, S1d) or antigen (Supplemental Fig. S1b) was detected in the nasal epithelia of the hamsters at 14 dpi.

### Comparison of Viral RNA Distribution in Lungs and Brains of K18-hACE2 Tg Mice, hACE2 Tg Mice, and Hamsters

**Figures 10–13.**
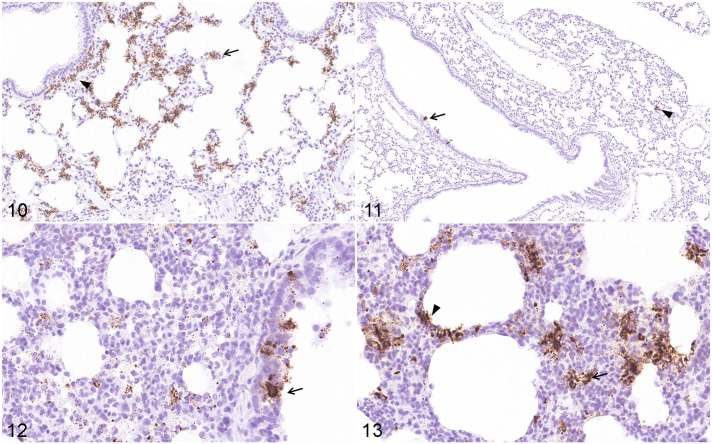
Distribution of SARS-CoV-2 RNA, lung. In situ hybridization for SARS-CoV-2. **Figure 10**. K18-hACE2 Tg mouse, 3 dpi. Viral RNA is abundant in alveolar epithelial cells (arrow) and inflammatory cells (arrowhead) in the alveolar septum and adjacent to airways. **Figure 11**. hACE2 Tg mouse, 5 dpi. Scattered viral RNA is detected in the epithelium of a bronchus (arrow) and pneumonocytes (arrowhead). **Figures 12, 13**. Syrian hamster, 7 dpi. Abundant viral RNA is detected in the epithelium of a bronchus (Fig. 7c, arrow) and in pneumonocytes (Fig. 7d, arrowhead), and infiltrating inflammatory cells (Fig. 7d, arrow) in the alveolar septum. Abbreviations: SARS-CoV-2, severe acute respiratory syndrome coronavirus-2; hACE2, human angiotensin-converting enzyme 2; dpi, days post-infection.

**Figures 14–17.**
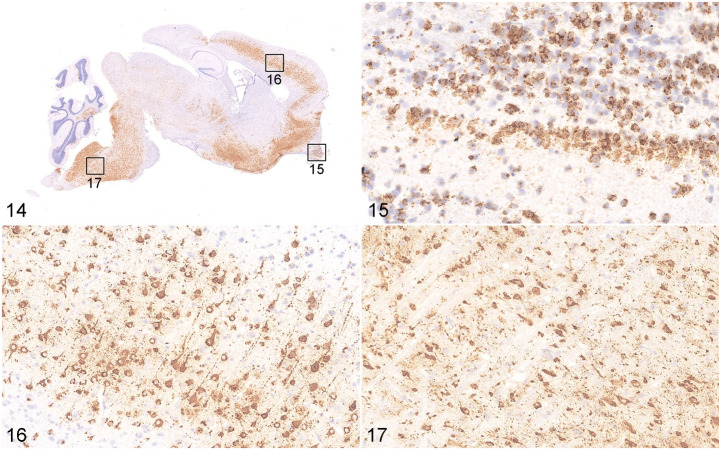
SARS-CoV-2 infection, brain, K18-hACE2 Tg mouse, 3 days post-infection. In situ hybridization (ISH). Viral RNA is massively distributed in the brain (Fig. 14), including a large amount in the olfactory bulb (Fig. 15), the cerebral cortex (Fig. 16), and the brainstem (Fig. 17). Abbreviations: SARS-CoV-2, severe acute respiratory syndrome coronavirus-2; hACE2, human angiotensin-converting enzyme 2; Tg, transgenic.

Using ISH, we confirmed that SARS-CoV-2 viral RNA was distributed in distinct locations and cell types of the lungs of K18-hACE2 Tg mice (3 dpi), hACE2 Tg mice (5 dpi), and hamsters (7 dpi), when the most severe clinical signs occurred (data not shown). By ISH, SARS-CoV-2 viral RNA was massively distributed throughout the lungs (Fig. 10) and brain (Fig. 14) of the K18-hACE2 Tg mice, and scattered viral RNA was detected in the epithelium of a bronchus and pneumonocytes of the hACE2 Tg mice (Fig. 11), whereas a moderate amount of SARS-CoV-2 RNA was detected in the lungs of the hamster model (Figs. 12, 13). Moreover, there was a distinct distribution pattern of SARS-CoV-2 viral RNA in each model. In the K18-hACE2 Tg mice, SARS-CoV-2 viral RNA was massively distributed in the pulmonary alveolar epithelial cells and inflammatory cells infiltrating the alveolar septum of the lungs, as well as in the granular cell layer of cerebrum and cerebellum (Fig. 14). A large amount of positive labeling was also detected in the olfactory bulb (Fig. 15), cerebral cortex (Fig. 16), and brainstem (Fig. 17), and sporadic viral-RNA-positive neurons were found in the pyriform (Purkinje) cell layer of the cerebellum and the hippocampus. In the hACE2 Tg mouse model, only sporadic SARS-CoV-2 viral RNA was detected in the epithelium of the bronchi and pneumocytes. In the lungs of the hamster model, abundant viral RNA was detected in the epithelium of the bronchi, pneumocytes, and inflammatory cells infiltrating the alveolar septum. No SARS-CoV-2 viral RNA was detected in the brains of the mock K18-hACE2 Tg mice, hACE2 Tg mice, and hamsters (data not shown).

## Discussion

SARS-CoV-2 enters the host through the respiratory tract, and the alveolar epithelial cells, vascular endothelial cells, and alveolar macrophages are among their first targets for viral entry.^8,10,15,18^ SARS-CoV-2 can efficiently infect tissues in the upper respiratory tract, such as nasopharyngeal and oropharyngeal tissues, possibly due to its high affinity for ACE2, which is expressed on human nasal and oral cells.^14,19,34,42^

Animal models for SARS-CoV-2 are needed to evaluate the effectiveness of countermeasures against SARS-CoV-2 infection. Similar to the abovementioned studies on human clinical cases, our results from animal models were also supportive of tissue tropism to upper respiratory epithelia. In this study, we analyzed the histopathological features of the nasal passages, lung, and brains in experimentally SARS-CoV-2-infected K18-hACE2 Tg mice, hACE2 Tg mice, and hamsters, at 3 dpi, 5 dpi, and 7 dpi, respectively, when the most severe stages of pneumonia were seen. Our results showed that SARS-CoV-2 viral RNA was detectable in the olfactory bulb of K18-hACE2 Tg mice. In agreement with our findings, infectious virus has been detected in the respiratory tract and the central nervous system, which suggests that SARS-CoV-2 initially targets the respiratory tract, but there can be secondary central nervous system involvement.^25,35^ These findings suggest that the virus may spread from the upper respiratory tract to the brain after intranasal inoculation in K18-hACE2 Tg mice.

Moreover, we compared the distribution of SARS-CoV-2 viral RNA in the squamous, respiratory, and olfactory epithelia of the nasal cavities and found that the nasal passages of the 3 models manifested different degrees of lesions, but the changes were generally focused at the olfactory epithelia. Comparing the different lesions among the 3 rodent models, rhinitis lesions were diagnosed as moderate in the K18-hACE2 Tg mice, mild in hACE2 Tg mice, and severe in hamsters. On the basis of our observation, the nasal passages of the K18-hACE2 Tg mice exhibited moderately severe lesions, including mucus exudation, focal irregularity, and necrosis of the olfactory epithelium. The nasal passages of hamsters exhibited severe degrees of exudative rhinitis, including degeneration and lymphocyte infiltration in the respiratory epithelia, and disordered arrangement, necrosis, multifocal exfoliation, massive inflammatory exudate, and inflammatory cells in the olfactory epithelium. Previous studies on hamsters also demonstrated that massive damage, apoptosis, and severe destruction of the olfactory epithelium occurred after intranasal inoculation with SARS-CoV-2, resulting in a major loss of the cilia necessary for odor detection.^2,37^ In this study, we found that microscopic lesions in the olfactory epithelium of the hamster model returned to normal by 14 dpi. A previous study also demonstrated that olfactory epithelial damage related to SARS-CoV-2 infection may be fully reversible.^30^ Moreover, similar to our study, there was no virus detected in the olfactory bulbs of hamster models,^2,37^ in contrast to what we found with K18-hACE2 Tg mice. In contrast to the K18-hACE2 Tg mice and the hamsters, our observations indicated that the olfactory epithelia of hACE2 Tg mice remained intact.

Viral RNA was detected in different parts of the olfactory mucosa by ISH. In K18-hACE2 Tg mice, a large amount of viral RNA was detected in the lamina propria underlying the olfactory epithelium. In hamsters, massive amounts of viral RNA were detected in the exfoliated cells within the inflammatory exudate, but only a small amount was focally detected in the epithelium and lamina propria of the olfactory mucosa. In addition, it is worth noting that SARS-CoV-2 nucleocapsid protein was present in the neurons of the olfactory bulb in our K18-hACE2 Tg mouse model, but not in hACE2 Tg mice or hamsters. Similar to these results, several human autopsy reports have documented the presence of SARS-CoV-2 RNA in brain tissues.^26,27^ Coincidently, a previous study on SARS-CoV-2-infected K18-hACE2 mice also reported SARS-CoV-2 infection of the nasal turbinate, eyes, and olfactory bulb, indicating that the virus may enter the brain by the nasal route after intranasal infection.^20^ Our observations imply that the lamina propria underlying the olfactory epithelium may play an important role in the infection of the olfactory nerves, as an infection route from the nose to the brain in K18-hACE2 Tg mice.

In conclusion, we evaluated the pathological characteristics of the nasal passages of K18-hACE2 Tg mice, hACE2 Tg mice, and Syrian hamsters infected with SARS-CoV-2. The models mimic the different outcomes of SARS-CoV-2 infection in humans and provide a variety of tools to develop our understanding of the pathogenesis of COVID-19. They might also be suitable models for aiding in the development of effective medications and prophylactic treatments for SARS-CoV-2 infection.

## Supplemental Material

sj-pdf-1-vet-10.1177_03009858211071016 – Supplemental material for Comparative pathology of the nasal epithelium in K18-hACE2 Tg mice, hACE2 Tg mice, and hamsters infected with SARS-CoV-2 Click here for additional data file. Supplemental material, sj-pdf-1-vet-10.1177_03009858211071016 for Comparative pathology of the nasal epithelium in K18-hACE2 Tg mice, hACE2 Tg mice, and hamsters infected with SARS-CoV-2 by Pin Yu, Wei Deng, Linlin Bao, Yajin Qu, Yanfeng Xu, Wenjie Zhao, Yunlin Han and Chuan Qin in Veterinary Pathology
