# Supplementary material for: Comparative pathology of the nasal epithelium in K18-hACE2 Tg mice, hACE2 Tg mice, and hamsters infected with SARS-CoV-2
Source: Vet Pathol. 2022 Jan 29;59(4):602–12. doi: 10.1177/03009858211071016 (PMC9208069; doi:10.1177/03009858211071016)
Supplement: sj-pdf-1-vet-10.1177_03009858211071016 – Supplemental material for Comparative pathology of the nasal epithelium in K18-hACE2 Tg mice, hACE2 Tg mice, and hamsters infected with SARS-CoV-2 [file sj-pdf-1-vet-10.1177_03009858211071016.pdf]

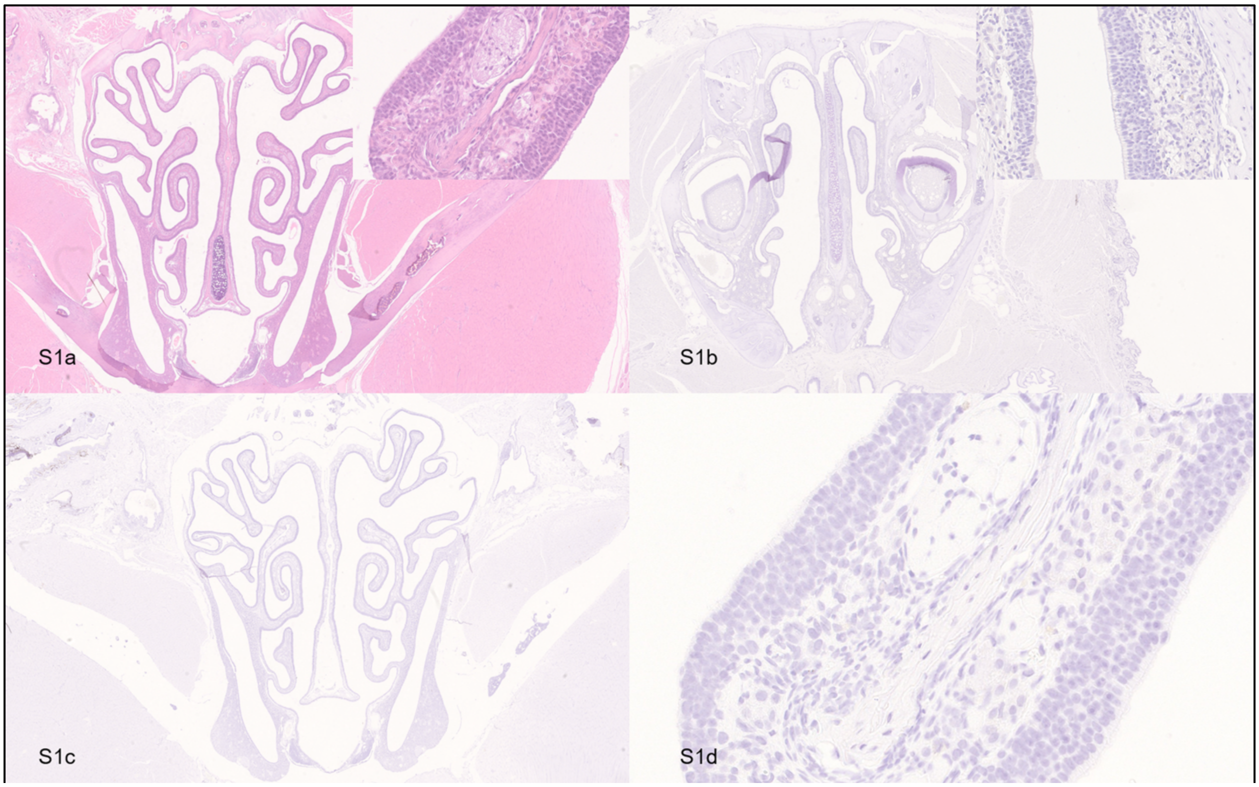

Supplemental Figure S1. Lesion recovery, nasal mucosa, Syrian hamster, 14 dpi. Fig. S1a. A mild disordered arrangement of the olfactory sensory cells (arrow) is focally observed. Hematoxylin-eosin (HE). Fig. S1b. No SARS-CoV-2 antigen is detected in the olfactory epithelium. Immunohistochemistry (IHC) for nucleocapsid protein. Fig. S1c. No SARS-CoV-2 RNA is detected in the olfactory epithelium. In situ hybridization (ISH). Fig. S1d. Focal magnification of Fig. S1c shows absence of labeling for SARS-CoV-2 viral RNA in the olfactory epithelium. ISH.

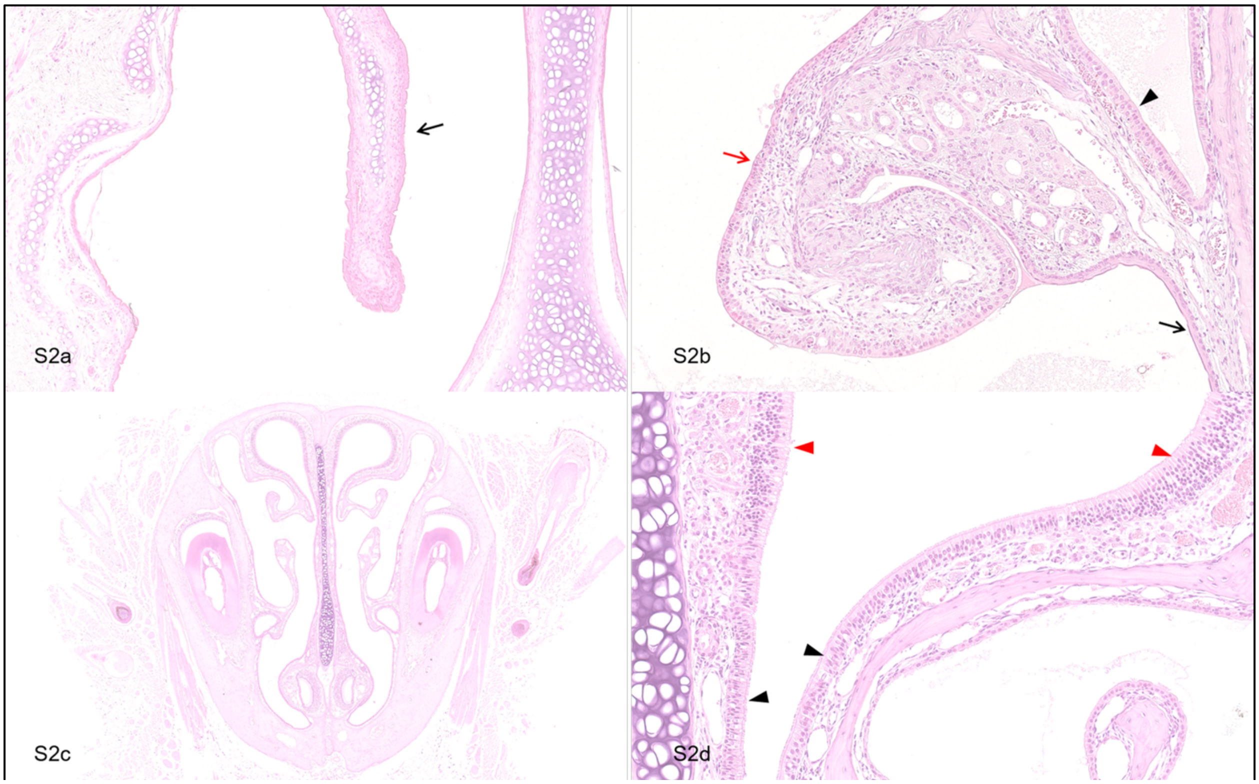

Supplemental Figure S2. Normal histological features, nasal mucosa, Syrian hamster and K18-hACE2 Tg mouse. Fig. S2a. Black arrow shows squamous epithelium in the nasal passage of Syrian hamster. Hematoxylin-eosin (HE). Fig. S2b. In the nasal passage of Syrian hamster, black arrow shows squamous epithelium. Red arrow shows transitional epithelium. Black arrowhead shows respiratory epithelium. HE. Fig. S2c. Nasal passage of K18-hACE2 Tg mouse is shown. HE. Fig. S2d. In the nasal passage of K18-hACE2 Tg mouse, black arrowhead shows respiratory epithelium. Red arrowhead shows olfactory epithelium. HE.

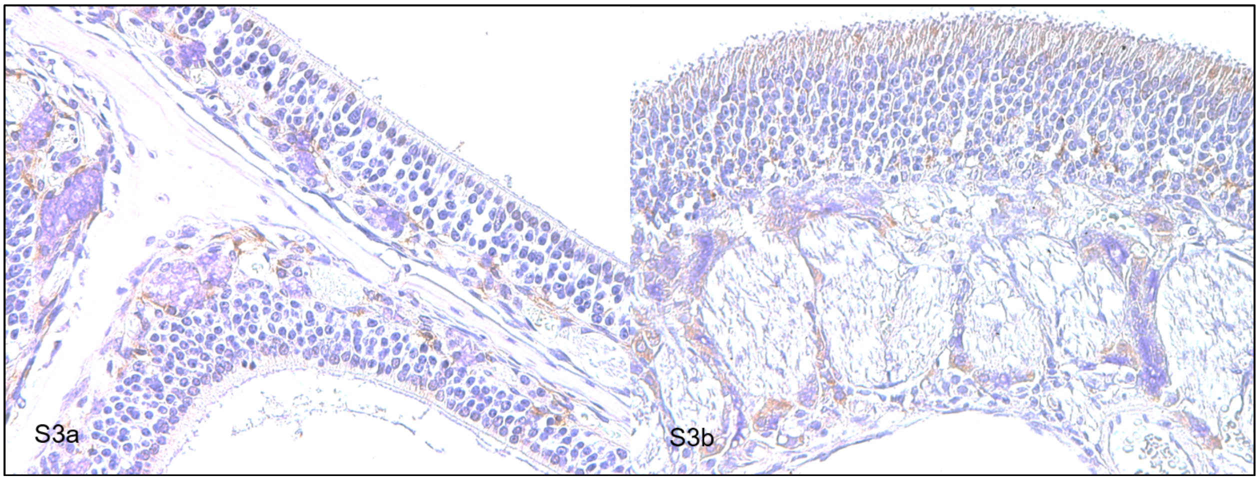

Supplemental Figure S3. Normal distribution of macrophages, nasal mucosa, K18-hACE2 Tg mouse and Syrian hamster. Fig. S3a. Normal diffuse distribution of F4/80-positive macrophages in lamina propria of the nasal passage of mock K18-hACE2 Tg mouse. Immunohistochemistry (IHC) for F4/80. Fig. S2b. Normal diffuse distribution of F4/80-positive macrophages in lamina propria of the nasal passage of mock Syrian hamster. IHC for F4/80.
